# Standardizing care for agitation in Alzheimer's disease, results from a randomized controlled trial of an integrated care pathway versus usual care – the StaN trial

**DOI:** 10.1002/alz.71610

**Published:** 2026-07-27

**Authors:** Sanjeev Kumar, Amer M. Burhan, Sarah Colman, Li Chu, Simon Davies, Peter Derkach, Sarah Elmi, Philip Gerretsen, Ariel Graff‐Guerrero, Kyle Greenway, Maria Hussain, Zahinoor Ismail, Donna Kim, Linda Krisman, Clement Ma, Rola Moghabghab, Benoit H. Mulsant, Bruce G. Pollock, Soham Rej, Aviva Rostas, Nicole Schoer, David L. Streiner, Lisa Van Bussel, Tarek K. Rajji, the StaN Study Group, Saima Awan, Amer M. Burhan, Sarah Colman, Susmita Chandramouleeshwaran, Samira Choudhury, Li Chu, Steve Crawford, Simon Davies, Peter Derkach, Sarah Elmi, Philip Gerretsen, Ariel Graff‐Guerrero, Maria Hussain, Zahinoor Ismail, Donna Kim, Dunja Knezevic, Linda Krisman, Sanjeev Kumar, Paul Kurdyak, Lillian Lourenco, Ashley Melichercik, Rola Moghabghab, Benoit H. Mulsant, Vasavan Nair, Claire de Oliveira, Shima Ovaysikia, Bruce G. Pollock, Tarek K. Rajji, Soham Rej, Aviva Rostas, Dallas Seitz, Jyll Simmons, David Streiner, Christopher Uranis, Lisa Van Bussel, Erica Vieira, Vincent L. Woo, Shadi Zarei

**Affiliations:** ^1^ Department of Psychiatry Temerty Faculty of Medicine University of Toronto Toronto Ontario Canada; ^2^ Division of Geriatric Psychiatry Centre for Addiction and Mental Health Toronto Ontario Canada; ^3^ Toronto Dementia Research Alliance Toronto Ontario Canada; ^4^ Department of Geriatric Psychiatry Ontario Shores Centre for Mental Health Sciences Whitby Ontario Canada; ^5^ West Park Health Care Centre Toronto Ontario Canada; ^6^ Ukrainian Canadian Care Centre Toronto Ontario Canada; ^7^ Department of Psychiatry McGill University Montreal Quebec Canada; ^8^ Department of Psychiatry Queen's University Kingston Ontario Canada; ^9^ Department of geriatric psychiatry Providence Care Hospital Kingston Ontario Canada; ^10^ Departments of Psychiatry, Clinical Neurosciences, Community Health Sciences, and Pathology & Laboratory Medicine University of Calgary Calgary Alberta Canada; ^11^ Faculty of Health and Life Sciences, NIHR Biomedical Research Unit University of Exeter Exeter UK; ^12^ Division of Biostatistics, Dalla Lana School of Public Health University of Toronto Toronto Ontario Canada; ^13^ The Royal Hospital Ottawa Ontario Canada; ^14^ Department of Psychiatry and Behavioural Neurosciences McMaster University Hamilton Ontario Canada; ^15^ Department of Psychiatry Western University London Ontario Canada; ^16^ Department of Psychiatry UT Southwestern Medical Center Dallas Texas USA

**Keywords:** agitation in dementia, Alzheimer's disease, behavioral and psychological symptoms of dementia, integrated care pathway, neuropsychiatric symptoms, non‐pharmacological interventions, polypharmacy

## Abstract

**BACKGROUND:**

Adherence to treatment guidelines for agitation in dementia is suboptimal and inconsistent. We designed and evaluated an Integrated Care Pathway (ICP) for the management of agitation in dementia.

**METHODS:**

This was a double‐blind randomized controlled trial at 12 inpatient units and long‐term‐care homes (LTCHs) across Canada. Participants were randomized 1:1 to the ICP or treatment‐as‐usual (TAU). Primary outcomes were Cohen Mansfield Agitation Inventory (CMAI) and psychotropic polypharmacy at 12 weeks.

**RESULTS:**

We randomized 185 participants (93 inpatients, 92 in LTCHs). For CMAI, there were no significant time‐by‐treatment‐group interactions among inpatients (F_4, 299.3 _= 1.7, *p* = 0.14) or LTCH residents (F_4, 296.0 _= 0.87, *p* = 0.48). For polypharmacy, there were significant time‐by‐treatmentgroup interactions among both inpatients ($\chi_{7}^{2}$= 15.3, *p* = 0.032) and LTCH residents ($\chi_{7}^{2}$= 30.0, *p* < 0.001), with lower rates of polypharmacy in the ICP group at certain time points, but not at week 12.

**CONCLUSIONS:**

Standardizing care for agitation in dementia may result in lesser polypharmacy without affecting efficacy. Future studies should assess the ICP in the broader community and outpatients.

## Highlights

- Adherence to guidelines for the management of agitation in dementia is poor, and inappropriate use of psychotropic medications is common.
- We developed and evaluated an Integrated care pathway  (ICP) that provides an algorithmic and measurement‐based care model for the management of agitation in dementia.
- This multisite randomized double‐blind controlled trial compared the ICP with usual care in inpatient and long‐term care homes affiliated with academic hospitals in Canada.
- There were no differences in clinical outcomes in terms of agitation symptoms between the ICP and usual care; however, there was lower polypharmacy use in the ICP group during the trial, but again, no differences at the end of the trial at 12 weeks.
- This trial highlights that it may be feasible to manage agitation in dementia using the ICP and that future studies may evaluate the ICP in community settings outside of academic centers.

## 1 INTRODUCTION

By 2050, more than 130 million people worldwide will be living with dementia, with the most common form being Alzheimer's disease (AD).^1^ The majority of patients with AD experience neuropsychiatric symptoms (NPSs), specifically agitation, that imposes a significant burden on patients, their families, caregivers, and healthcare systems.^2^, ^3^ Agitation is also the leading cause of hospitalizations and transfer to long‐term‐care homes (LTCHs).^3^, ^4^

Agitation is challenging to manage, with recent evidence identifying key barriers including an overuse of physical and chemical restraints and an underuse of non‐pharmacological interventions.^5^ Agitation is often treated with antipsychotic medications, which can be associated with serious adverse events, including falls, cerebrovascular accidents, and death.^6^ Further, psychotropic polypharmacy (use of more than one psychotropic medication) is common in patients with dementia, with estimates ranging from 25% to 79% depending on the specific population and setting.^7^, ^8^, ^9^, ^10^, ^11^ While previous studies did not define polypharmacy based on specific indications, the literature suggests that NPSs such as agitation are a significant contributor to the psychotropic polypharmacy in this population.^12^, ^13^, ^14^ Psychotropic polypharmacy in patients with dementia has seen an increase,^8^, ^15^ despite the reduction in antipsychotic prescribing following the black box warnings regarding their use in dementia.^16^ Psychotropic polypharmacy is associated with adverse outcomes in older adults, including increased rates of falls, hospitalizations, delirium, mortality, and rapid cognitive decline.^17^, ^18^, ^19^, ^20^, ^21^, ^22^, ^23^ Psychotropic polypharmacy in dementia may be related to suboptimal dosing of psychotropics,^24^ lack of standardized treatment approaches,^25^, ^26^, ^27^, ^28^, ^29^ and the underutilization of non‐pharmacological interventions.^30^ Evidence‐based guidelines for the treatment of agitation have attempted to address these problems^31^, ^32^, ^33^; however, poor adherence has limited the impact of guidelines alone.^34^

To address these issues, we developed and implemented an algorithm‐based Integrated Care Pathway (ICP),^35^ which standardizes the treatment of agitation in AD using a structured approach integrating non‐pharmacological and psychotropic interventions with measurement‐based decisions.^35^, ^36^ Algorithmic treatments have been shown to improve treatment outcomes for both physical^37^ and mental disorders.^38^, ^39^, ^40^ Algorithmic treatments use a sequential and measurement‐based approach to interventions allowing for standardization of when to start an intervention, what target to reach, and for how long to treat. Most treatment algorithms for agitation in dementia lack this specific sequential and measurement‐based care approach.^41^, ^42^ In a small pilot study that evaluated the implementation of our ICP on a geriatric psychiatric inpatient unit, it increased efficiency of care and reduced psychotropic polypharmacy.^14^ Building on this pilot study, we conducted a 12‐week double‐blind randomized controlled trial (RCT) to compare the clinical efficacy, impact on psychotropic polypharmacy, and safety of the ICP and treatment as usual (TAU) in both inpatient and LTCH settings. Our co‐primary hypotheses were that, compared to TAU, (1) the ICP would result in reduced agitation, as assessed using the Cohen–Mansfield Agitation Inventory (CMAI) at week 12, and (2) the ICP would result in a lower proportion of participants on psychotropic polypharmacy for agitation at week 12. Our exploratory hypotheses were that, compared to TAU, the ICP would result in shorter time to response for agitation, a lower global burden of NPS, a lower rate of falls, better health‐related quality of life, and lesser caregiver burden.

**RESEARCH IN CONTEXT**

1. **Systematic review**: The authors reviewed the literature using PubMed and other resources. While inappropriate medication use is common, no existing randomized controlled trials compared algorithmic and measurement‐based approaches with usual care for agitation in dementia.
2. **Interpretation**: Our findings show that while the ICP to manage agitation in dementia was not superior to usual care in terms of reduction of symptoms in tertiary‐level hospital inpatient units and long‐term care homes, it may result in lesser polypharmacy in both these settings.
3. **Future directions**: This manuscript presents a robust multisite trial in a unique and challenging environment and highlights the need for potentially conducting such trials in the future in community settings to assess the efficacy of the ICP to manage agitation related to dementia. This might improve clinical outcomes and reduce adverse effects from inappropriate use of medications in elderly with dementia and agitation.

## 2 METHODS

### 2.1 Overview

Standardizing Care for Neuropsychiatric Symptoms and Quality of Life in Dementia (StaN trial, ClinicalTrials.gov No. NCT03672201), a double‐blind RCT, was conducted in five psychiatry inpatient units and seven LTCHs affiliated with academic centers across three Canadian provinces: Alberta, Ontario, and Quebec. The study was approved by research ethics boards at all sites, and written informed consent was obtained from participants or their substitute decision makers before any study procedures.

### 2.2 Participants

Participants were included if they were 50 years or older, were admitted to one of the five inpatient units or were residing in one of the seven LTCHs, had a clinical diagnosis of Major Neurocognitive Disorder (dementia) due to possible or probable AD based on the *Diagnostic and Statistical Manual of Mental Disorders*, 5th Ed. (DSM‐5) criteria^43^ and were experiencing clinically significant agitation as assessed using the International Psychogeriatric Association (IPA) provisional consensus clinical and research definition.^44^ Participants were excluded if they had DSM‐5 diagnoses other than major neurocognitive disorder that could significantly contribute to their agitation (e.g., delirium, unstable bipolar disorder) or if they were otherwise medically unstable as determined by a study investigator.

### 2.3 Randomization

Participants were randomized 1:1 to either the ICP or TAU. Randomization was under the supervision and control of study biostatistician. A software system using balanced random assignments to treatment was used to generate a treatment assignment schedule in a random permuted block design. The trial was double‐blinded: participants, their substitute decision makers, and the outcome assessors (Assessment Research Analysts [RAs] were blinded to the treatment assignment. Participants’ clinical teams, the Intervention RAs, and the study physicians were not masked to the assignment.

### 2.4 Interventions

Participants in the ICP group underwent a stepwise intervention for 12 weeks, which consisted of (1) a clean‐up phase during which unnecessary or ineffective medications for agitation were discontinued, (2) a concomitant structured behavioral interventions phase during which a comprehensive behavioral assessment was completed using a standardized interdisciplinary approach and a personalized behavioral intervention plan was developed and implemented,^36^ and (3) a sequential psychotropic intervention phase guided by measurement‐based care that typically started, if needed, after 3 weeks of the behavioral intervention phase. The behavioral interventions were continued during this phase.^35^ Each of the three ICP components were implemented by the clinical teams based on the guidance of the research team, typically communicated by the Intervention RAs. The Intervention RAs at each site also collected the necessary clinical information about the participants in the ICP group from their health records and the clinical teams and used this information to rate changes in participants’ agitation using the modified Clinical Global Impression of Change (CGIC). The CGIC is a 7‐point rating scale, ranging from 1 (very much improved) to 7 (very much worse). This unmasked CGIC was used to implement measurement‐based care as laid out in the ICP.^35^, ^36^ In brief, participants were treated by modifying or switching interventions until they achieved a CGIC of 1 (very much improved) or 2 (much improved).^45^ Once they achieved one of these two scores, participants were maintained on the ICP interventions (psychotropic medications and ongoing behavioral interventions) that were in place when this score was attained. If a participant's CGIC score worsened afterward, then changes in the interventions were implemented following the ICP.

All participants were discussed during weekly research meetings for the entire duration of the intervention (12 weeks). At these meetings the clinical status of participants in the ICP group was reviewed and recommendations to the clinical teams were formulated regarding the next ICP steps. These recommendations were communicated to the clinical teams by the research team, and the clinical teams were free to follow these recommendations or not as they deemed appropriate. The clinical teams treated participants in the TAU group as per standard of care, including pharmacological and behavioral interventions as deemed appropriate; the research team did not make any recommendations, nor did they restrict the clinical teams from implementing any intervention.

### 2.5 Assessments

Outcomes were assessed in both treatment groups at the same time points (Figure S1) by trained research analysts (Assessment RAs), masked to the intervention. The first primary outcome – agitation – was assessed using the 29‐item CMAI,^45^ at baseline and at weeks 1, 3, 8, and 12 after randomization. For CMAI, the Assessment RA recorded the frequency of each behavior during the previous 2 weeks based on the caregiver's input. The behaviors were rated from 1 (never) to 7 (Several times an hour). To calculate the total score, we assigned a score of 0 to all behaviors scored 1 and then calculated the sum of all behaviors. Thus, the possible range for the total score was 0 to 203. The rationale for not including scores of 1 was to allow better detection of change in behaviors.

The other primary outcome – psychotropic polypharmacy for the treatment of agitation – was assessed using medication records at baseline, end of weeks 1, 3, 4, and then every 2 weeks until week 12. Psychotropic polypharmacy was defined as the use of two or more scheduled psychotropic medications for the treatment of agitation at a given time.

In addition to the unmasked CGIC, completed by the unmasked Intervention RAs only in the ICP treatment group to guide the implementation of the ICP (see above), a masked CGIC was completed for all participants by the masked Assessment RAs and used as an outcome measure at the end of week 1 and every 2 weeks thereafter. The number of falls was recorded every 2 weeks throughout the study. The following measures were completed at weeks 1, 3, and 12: Abnormal Involuntary Movement Scale (AIMS),^46^ Simpson Angus Scale (SAS),^47^ and Barnes Akathisia Rating Scale (BAS)^48^ were completed to assess medication‐related adverse effects. The Pain Assessment in Advanced Dementia (PAINAD)^49^ was used to assess pain, and the Morse Fall Scale (MFS)^50^ was used to quantify fall risk. The Neuropsychiatric Inventory‐Clinician rated (NPI‐C)^51^ was completed to assess global burden of NPSs. The quality of life was assessed with the Alzheimer's Disease‐Related Quality of Life (ADRQL)^52^ and the Caregiver burden with the Zarit Burden Interview (ZBI).^53^ Stage of dementia was ascertained using the Functional Assessment Staging Tool (FAST).^54^ Cognitive assessments were conducted using the Montreal Cognitive Assessment (MoCA),^55^ or the Severe Cognitive Impairment Rating Scale (SCIRS)^56^ if the participant scored <5 on the MoCA. Burden of physical comorbidity was assessed using the Cumulative Illness Rating Scale‐Geriatric (CIRS‐G).^57^ Adverse events were recorded throughout the study and reported immediately as needed in accordance with regulatory procedures.

The study was conducted from November 2018 to February 2021, during which the COVID‐19 pandemic affected certain study activities. During the pandemic, to decrease the risk of infection to study participants, the research team collected participant data virtually, and all in‐person meetings were switched to virtual meetings. Accordingly, most of the assessments (CMAI, NPI‐C, CGIC, FAST, ZBI, ADRQL, CIRS‐G, and MFS) were conducted virtually using video or phone interviews with staff or caregivers, while other assessments (MoCA, SCIRS, AIMS, SAS, BAS, and PAINAD) were suspended. The study recruited 48.1% (*n* = 89) of the participants in the pre‐pandemic period and 51.9% (*n* = 96) participants during the pandemic period.

### 2.6 Power analysis

For our primary hypothesis about the effect of the intervention on agitation, our pilot data on the ICP in 25 participants demonstrated a reduction in mean (standard deviation [SD]) CMAI total sum score from 57.5 (24.5) at baseline to 42.2 (18.4) upon completion of treatment. Assuming a two‐tailed α = 0.05, a mean (SD) change in CMAI total sum in TAU of 5.0 (18.4) points and 10% attrition, we estimated that 220 participants (with 55 in each group – ICP and TAU – in each setting – inpatient and LTCHs) would allow us to detect an effect size (Cohen's *d*) of 0.56 with 80% power in each setting. These estimates are consistent with changes in CMAI scores in other trials of agitation in dementia.^58^, ^59^ For our other primary hypothesis about the use of psychotropic polypharmacy, in our pilot study, only 3% of participants in the ICP were prescribed polypharmacy to treat agitation. Based on an estimate of psychotropic polypharmacy from the literature of 30% in TAU, we estimated that 220 participants would give us 97% power to detect a significant difference at α = 0.05 and β value of 0.2 within each setting.

### 2.7 Statistical analyses

We examined the distribution of variables using histograms and used log transformation or non‐parametric tests as needed in case of non‐normal distribution. We compared the demographic and clinical characteristics of the ICP and TAU groups using independent‐sample *t*‐tests or chi‐squared tests, as applicable. For our first primary outcome, we compared CMAI total sum scores in the ICP and TAU groups over time in each of the two settings (inpatient and LTCH) using linear mixed models (LMMs) with CMAI total sum score as the dependent variable, time as a repeated measure, the randomization group as the independent variable, and age, gender, stage of dementia (FAST score), and baseline CMAI total sum as covariates. We used an auto‐regressive covariance structure and a random intercept term to account for individual/group variability. We assessed the treatment effect using the time‐by‐intervention group interaction term. For our other primary outcome of psychotropic polypharmacy for the treatment of agitation, we used a generalized estimating equation (GEE) model with polypharmacy (yes/no) as a binary dependent variable, time as a repeated measure, and intervention group as the independent variable in each participant setting. The GEE model included age, gender, FAST score, and baseline polypharmacy status as covariates. We also assessed for treatment effect using the time‐by‐intervention group interaction term.

We built similar LMMs or generalized LMMs for other outcome variables such as ADRQL, NPI‐C, and ZBI, depending on the data distribution. Post‐hoc estimates were derived by comparing the randomization groups at each time point. Survival analysis was performed to compare the time to response (as assessed using CGIC < 3) between the ICP and TAU groups. Kaplan‐Meier survival curves were constructed to visualize the cumulative event probabilities over time for each group. The log‐rank test was used to assess the statistical significance of differences in the survival curves between the groups.

All analyses were done separately in the inpatient and LTCH cohorts and followed an intent‐to‐treat principle, with all randomized participants being included in the analyses. Participants who were lost to follow‐up were censored at the time they discontinued their participation. We conducted sensitivity analyses by including site as an independent variable. We examined the temporal patterns of dropouts and missing data in each arm to confirm that the data were missing at random. All analyses were performed using SPSS^®^ (IBM Corp., version 27.0).

## 3 RESULTS

### 3.1 Participant flow and baseline characteristics

**FIGURE 1:**
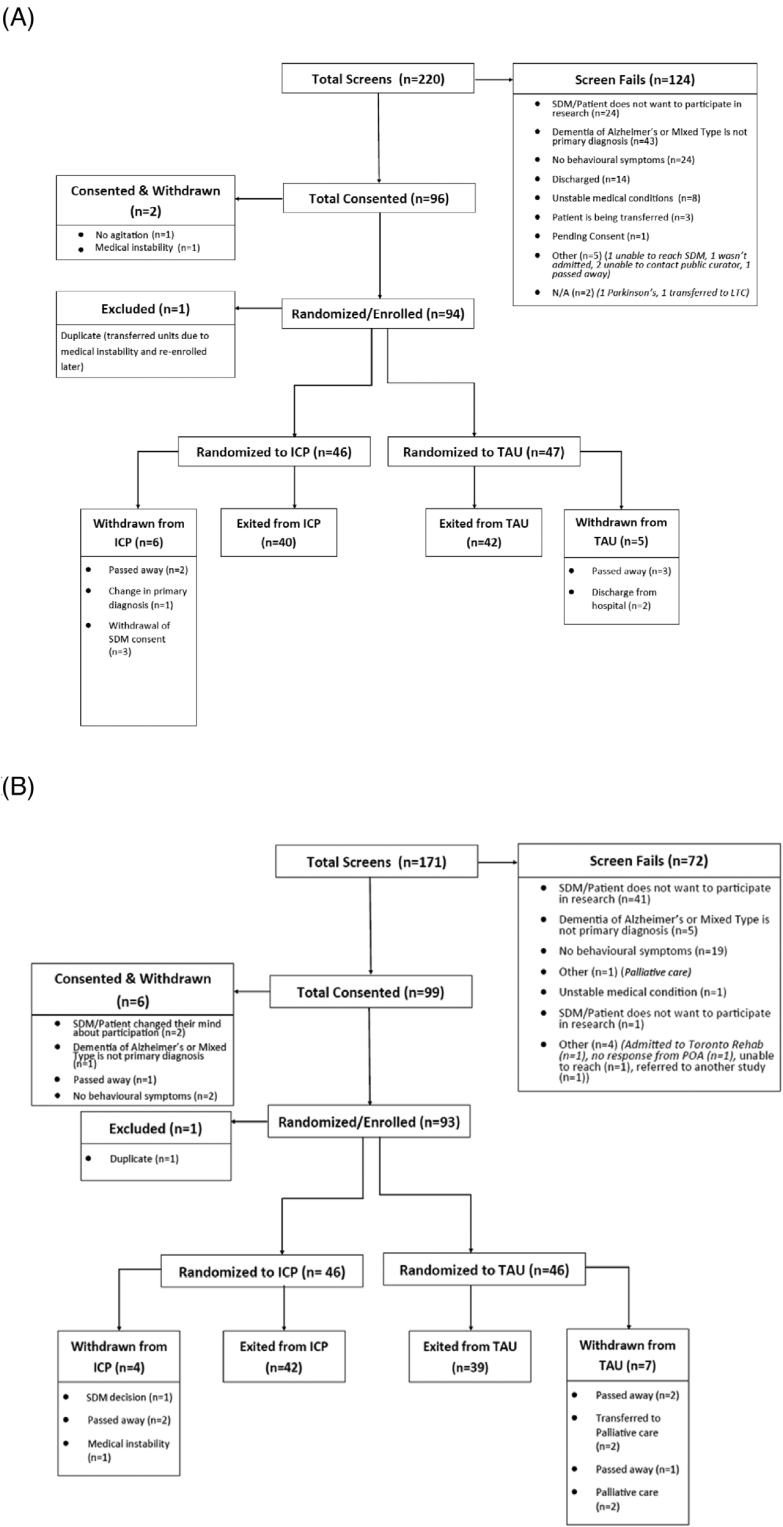
Consort chart showing flow of participants in study. (A) Inpatient. (B) Long‐term care (LTC). ICP, Integrated Care Pathway; SDM, substitute decision maker; TAU, treatment as usual.

Figure 1 (A and B) Flow of participants: Among the 93 inpatient participants, 46 were randomized to the ICP versus 47 to TAU; among the 92 LTCH participants, 46 were randomized to the ICP versus 46 to TAU. Table 1A and B summarizes the baseline characteristics of participants in the ICP and TAU groups. There were no significant differences between the groups in age, gender, or other demographic or clinical variables.

**TABLE 1A:** Baseline characteristics of inpatient patients by treatment group (ICP vs TAU).

	Inpatient				
Variable	Overall^a^	ICP^a^	TAU^a^	Test statistic^b^	*p* value^b^
	*N* = 93	*N* = 46	*N* = 47		
**Age**		75.2 (8.2)	74.7 (8.7)	*t* = 0.26	0.8
**Gender**				*χ* ^2^ = 0.53	0.5
Male	61.0 (65.6%)	28.0 (60.9%)	33.0 (70.2%)		
Female	32.0 (34.4%)	18.0 (39.1%)	14.0 (29.8%)		
**Education**	12.7 (4.2)	12.8 (4.7)	12.6 (3.8)	*t* = 0.23	0.8
**BMI**	25.3 (4.6)	25.6 (4.8)	24.9 (4.5)	*t* = 0.51	0.6
**Psychotropic polypharmacy excluding cognitive enhancers**				*χ* ^2^ < 0.001	>0.9
Yes	59.0 (64.1%)	29.0 (64.4%)	30.0 (63.8%)		
No	33.0 (35.9%)	16.0 (35.6%)	17.0 (36.2%)		
**Agitation medication polypharmacy**					0.061
Yes	47.0 (51.1%)	18.0 (40.0%)	29.0 (61.7%)		
No	45.0 (48.9%)	27.0 (60.0%)	18.0 (38.3%)		
**AIMS observed severity (N=52)**	1.0 (2.6)	1.6 (3.3)	0.4 (1.4)	*t* = 1.62	0.11
**BARS total score (N=53)**	0.9 (1.3)	1.0 (1.4)	0.8 (1.2)	*t* = 0.63	0.5
**CIRS‐G total score (N=88)**	8.2 (4.0)	8.5 (4.0)	8.0 (4.1)	*t* = 0.5	0.6
**CMAI total disruptiveness**	45.4 (13.8)	45.8 (15.0)	45.0 (12.5)	*t* = 0.26	0.8
**CMAI total frequency** ^c^	35.2 (23.2)	33.7 (26.3)	36.7 (19.8)	*t* = −0.62	0.5
**FAST stage number (N=89)**	9.1 (2.0)	9.2 (1.9)	9.1 (2.1)	*t* = 0.34	0.7
**MFS total score (N=89)**	46.8 (25.7)	50.2 (28.6)	43.6 (22.5)	*t* = 1.21	0.2
**MoCA total score (N=47)**	2.8 (4.8)	3.1 (5.4)	2.5 (4.3)	*t* = 0.42	0.7
**Number of medications indicated for agitation**	1.6 (1.1)	1.5 (1.2)	1.6 (1.1)	*t* = −0.45	0.7
**NPIC total sum**	50.3 (37.0)	47.5 (38.4)	52.9 (35.8)	*t* = −0.65	0.5
**PAINAD total score (N=53)**	1.4 (2.0)	1.5 (2.1)	1.3 (1.9)	*t* = 0.31	0.8
**SAS total score (N=53)**	4.6 (4.8)	4.1 (4.6)	5.2 (5.0)	*t* = −0.79	0.4
**SCIRS total score (N=37)**	9.6 (9.1)	10.1 (8.5)	9.2 (9.8)	*t* = 0.32	0.7
**ZBI total score (N=68)**	17.2 (17.4)	15.8 (16.4)	18.6 (18.4)	*t* = −0.67	0.5
**ADRQL total percentage (N=85)**	55.5 (17.6)	55.6 (17.8)	55.5 (17.6)	*t* = 0.03	>0.9

*Note*: Values are presented as mean (standard deviation) or *n* (%). *p* values are from independent‐sample *t*‐tests or Pearson's chi‐squared tests, as appropriate.

Abbreviations: AIMS, Abnormal involuntary movement scale; BARS, Barnes Akathisia Rating Scale; BMI, body mass index; CIRS‐G, Cumulative Illness Rating Scale–Geriatric; CMAI, Cohen–Mansfield agitation inventory; FAST, Functional Assessment Staging Tool (subtage scores for 6 and 7 were converted to numbers resulting in 1‐16 sequential stages); ICP, Integrated Care Pathway; MFS, Morse Fall Scale; MoCA, Montreal Cognitive Assessment; NPI‐C, Neuropsychiatric inventory–clinician rating scale; PAINAD, pain assessment in advanced dementia; SAS, Simpson–Angus scale; SCIRS, Severe Cognitive Impairment Rating Scale; TAU, treatment as usual; ZBI, Zarit Caregiver Burden Interview.

^a^ Mean (SD); *n* (%).

^b^ Welch two‐sample *t*‐test; Pearson's chi‐squared test.

^c^ CMAI total scores were calculated by recoding “never” responses as 0 and summing all items, resulting in a possible range of 0–203.

**TABLE 1B:** Baseline characteristics of LTCH patients by treatment group (ICP vs TAU).

		LTCH			
	Overall^a^	ICP^a^	TAU^a^	Test statistic^b^	*p* value^b^
Variable		*N* = 46	*N* = 46		
**Age**	85.9 (7.6)	86.0 (6.2)	85.7 (8.9)	*t* = 0.2	0.8
**Gender**				*χ* ^2^ = 0.2	0.7
Male	29.0 (31.5%)	16.0 (34.8%)	13.0 (28.3%)		
Female	63.0 (68.5%)	30.0 (65.2%)	33.0 (71.7%)		
**Education**	12.5 (4.1)	12.6 (4.0)	12.4 (4.2)	*t* = 0.21	0.8
**BMI**	25.5 (4.5)	26.0 (4.7)	25.1 (4.3)	*t* = 0.83	0.4
**Psychotropic polypharmacy excluding cognitive enhancers**				*χ* ^2^ < 0.001	>0.9
Yes	48.0 (52.2%)	24.0 (52.2%)	24.0 (52.2%)		
No	44.0 (47.8%)	22.0 (47.8%)	22.0 (47.8%)		
**Agitation medication polypharmacy**				*χ* ^2^ < 0.001	>0.9
Yes	25.0 (27.2%)	13.0 (28.3%)	12.0 (26.1%)		
No	67.0 (72.8%)	33.0 (71.7%)	34.0 (73.9%)		
**AIMS observed severity (N=62)**	1.1 (2.0)	1.1 (2.4)	1.1 (1.6)	*t* = 0.06	>0.9
**BARS total score (N=62)**	0.1 (0.4)	0.2 (0.5)	0.0 (0.2)	*t* = 1.57	0.12
**CIRS‐G total score (N=91)**	8.7 (4.1)	7.9 (4.1)	9.5 (4.0)	*t* = −1.89	0.061
**CMAI total disruptiveness**	49.7 (18.2)	47.5 (15.7)	51.8 (20.3)	*t* = −1.14	0.3
**CMAI total frequency** ^c^	37.4 (28.5)	33.4 (24.7)	41.4 (31.7)	*t* = −1.35	0.2
**FAST stage number (N=92)**	9.1 (2.3)	8.9 (2.5)	9.3 (2.0)	*t* = −0.82	0.4
**MFS total score (N=92)**	46.0 (24.4)	42.9 (23.4)	49.0 (25.3)	*t* = −1.2	0.2
**MoCA total score (N=52)**	2.7 (5.6)	2.8 (5.9)	2.5 (5.3)	*t* = 0.2	0.8
**Number of medications indicated for agitation**	1.0 (0.8)	1.0 (0.9)	1.0 (0.7)	*t* = 0.13	0.9
**NPIC total sum**	71.8 (73.3)	66.6 (69.1)	77.2 (77.7)	*t* = −0.68	0.5
**PAINAD total score (N=62)**	0.8 (1.5)	0.7 (1.2)	0.9 (1.8)	*t* = −0.5	0.6
**SAS total score (N=62)**	3.9 (4.9)	4.0 (5.0)	3.8 (4.9)	*t* = 0.15	0.9
**SCIRS total score (N=52)**	13.4 (10.1)	14.0 (9.9)	12.9 (10.5)	*t* = 0.39	0.7
**ZBI total score (N=89)**	22.8 (16.8)	23.2 (17.5)	22.5 (16.3)	*t* = 0.18	0.9
**ADRQL total percentage (N=91)**	66.2 (16.7)	69.1 (15.4)	63.3 (17.6)	*t* = 1.67	0.1

*Note*: Values are presented as mean (standard deviation) or *n* (%). *p* values are from independent‐sample *t*‐tests or Pearson's chi‐squared tests, as appropriate.

Abbreviations: AIMS, Abnormal Involuntary Movement Scale; BARS, Barnes Akathisia Rating Scale; BMI, body mass index; CIRS‐G, Cumulative Illness Rating Scale–Geriatric; CMAI, Cohen–Mansfield Agitation Inventory; FAST, Functional Assessment Staging Tool (subtage scores for 6 and 7 were converted to numbers resulting in 1‐16 sequential stages); ICP, Integrated Care Pathway; MFS, Morse Fall Scale; MoCA, Montreal cognitive assessment; NPI‐C, Neuropsychiatric inventory–clinician rating scale; PAINAD, pain assessment in advanced dementia; SAS, Simpson–Angus scale; SCIRS, Severe Cognitive Impairment Rating Scale; TAU, Treatment‐as‐usual; ZBI, Zarit caregiver burden interview.

^a^ Mean (SD); *n* (%).

^b^ Welch Two Sample *t*‐test; Pearson's chi‐squared test.

^c^ CMAI total scores were calculated by recoding “never” responses as 0 and summing all items, resulting in a possible range of 0–203.

### 3.2 Primary outcomes

**FIGURE 2:**
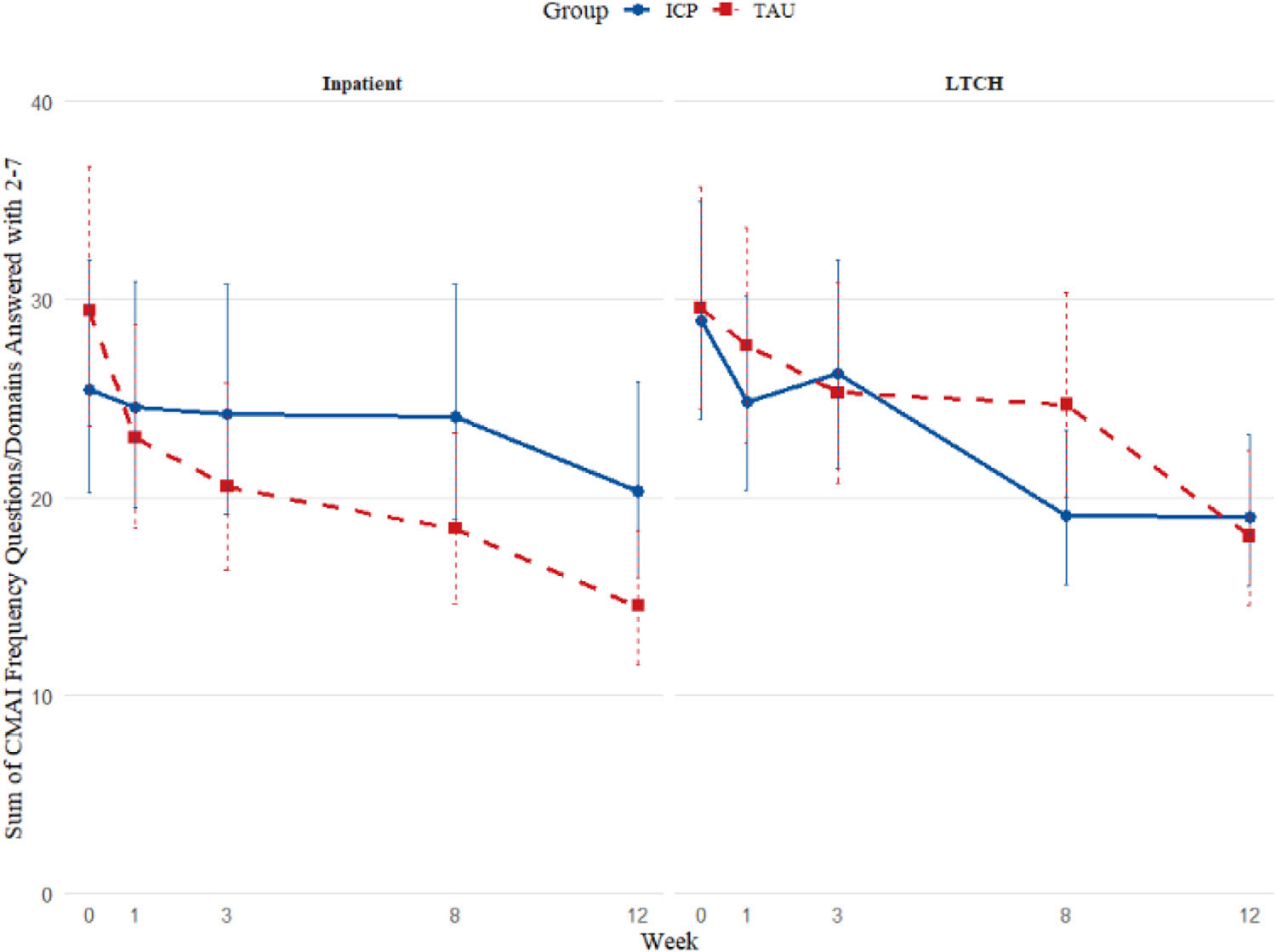
Estimated mean sum of Cohen‐Mansfield Agitation Inventory (CMAI) – frequency scores over time in inpatient and long‐term care plotted as ICP versus TAU in each setting. Note: Estimates were derived from a linear mixed model with CMAI frequency scores as the dependent variable; time, group (ICP vs TAU), and their interaction as fixed effects; and baseline CMAI, age, gender, and Functional Assessment Staging Tool score as covariates. The group × time interaction was not significant for inpatients (F = 1.7, *p* = 0.14) or LTCH residents (F = 0.87, *p* = 0.48). ICP, Integrated Care Pathway; TAU, treatment as‐usual; LTCH, long‐term‐care home. CMAI total scores were calculated by recoding “never” responses as 0 and summing all items, resulting in a possible range of 0–203.

Table 2A shows a summary of results from LMMs’ first primary outcome. The LMMs revealed no significant time‐by‐intervention group interaction over the 12‐week study among the inpatients (F_4, 299.3 _= 1.7, *p* = 0.14) or LTCH residents (F_4, 296.0 _= 0.87, *p* = 0.48) Further, there were no significant differences in CMAI total sum at week 12 between ICP and TAU for inpatients or LTCH residents (inpatient: ICP‐TAU mean difference = 0.334; 95% confidence interval [CI]: −0.002, 0.671; LTCH: ICP‐TAU mean difference = 0.051; 95% CI: −0.24, 0.35). Figure 2 displays the estimated marginal means (+95% CIs) for CMAI by group and time point.

**TABLE 2A:** Type III tests of fixed effects from linear mixed models for CMAI by setting (inpatient versus LTCH).

Covariate	Inpatient				LTCH			
	Numerator df	Denominator df	F statistic	*p* value	Numerator df	Denominator df	F statistic	*p* value
Age	1	143.385	0.030	0.864	1	154.902	3.067	0.082
Gender	1	146.297	5.013	0.027	1	155.679	1.982	0.161
Baseline Dementia Severity	1	149.469	4.586	0.034	1	150.833	0.002	0.969
Treatment group (ICP vs TAU)	1	139.405	2.146	0.145	1	144.776	0.664	0.417
Time point	4	299.487	6.035	<0.001	4	295.827	7.606	<0.001
Treatment group × Time interaction	4	299.341	1.739	0.141	4	295.961	0.874	0.480
Baseline agitation frequency (log‐transformed)	1	149.071	154.430	<0.001	1	149.347	471.049	<0.001

**FIGURE 3:**
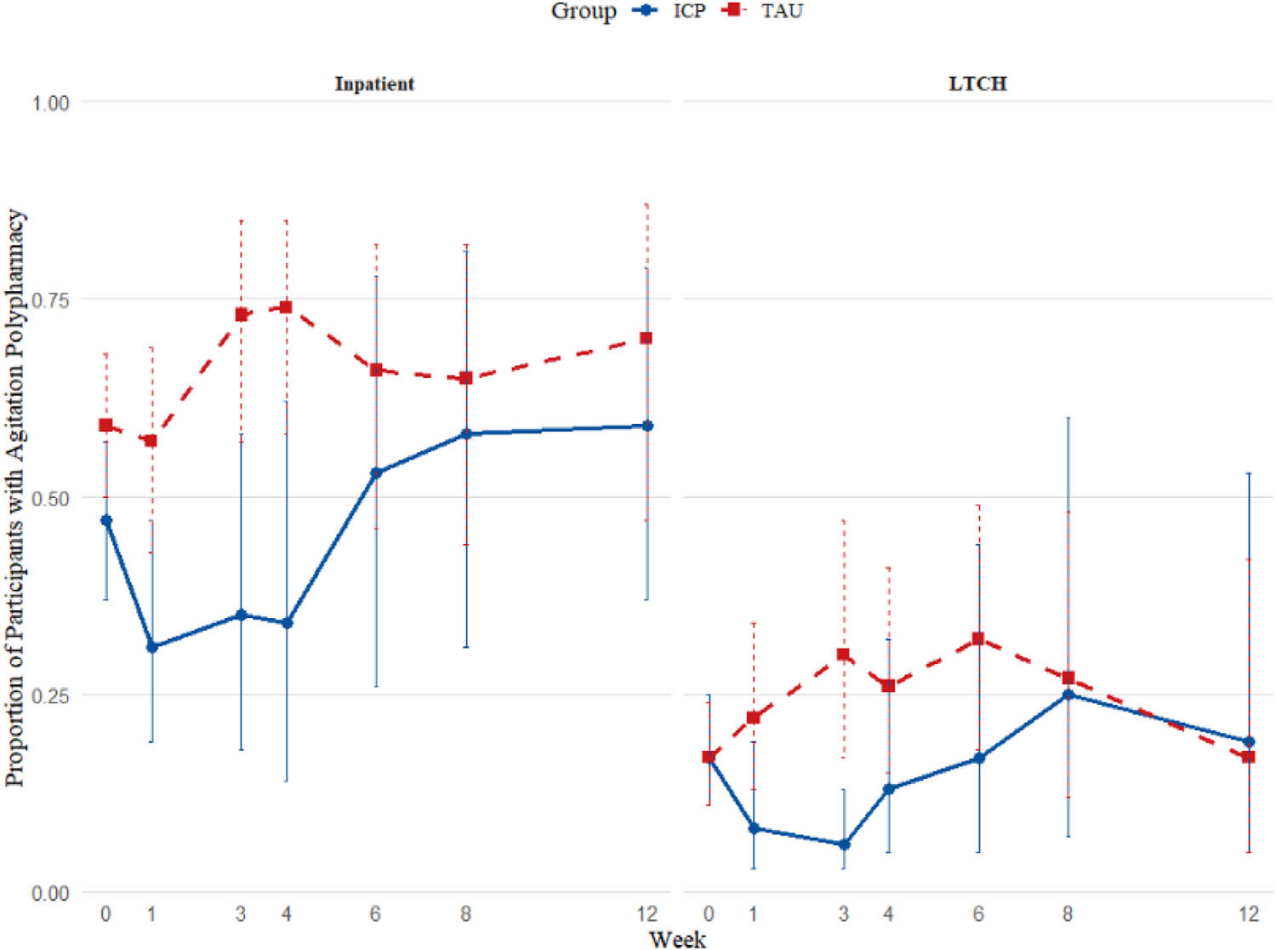
Estimated proportion of participants on psychotropic polypharmacy for treatment of agitation over time in inpatient and long‐term care plotted as ICP versus TAU in each setting. Note: Estimates are derived from a generalized estimating equation model with polypharmacy (defined as ≥2 medications prescribed for agitation) as the dependent variable; time, group (ICP vs TAU), and their interaction as predictors; and baseline polypharmacy, age, gender, and Functional Assessment Staging Tool score as covariates. The group × time interaction was significant for inpatients ($\chi_{7}^{2}$ = 15.3, *p* = 0.032, with post hoc significant differences at weeks 1, 3, and 4) and for LTCH residents ($\chi_{7}^{2}$ = 30.0, *p* < 0.001, with post hoc significant differences at weeks 1 and 3). ICP, Integrated Care Pathway; TAU, treatment as usual; LTCH, long‐term care home.

For our other primary outcome (psychotropic polypharmacy), there was a significant time‐by‐intervention group interaction for both the inpatients ($\chi_{7}^{2}$= 15.3, *p* = 0.032) and LTCH residents ($\chi_{7}^{2}$= 30.0, *p* < 0.001) during the 12 weeks of the study (Table 2B). However, there were no significant differences in either setting at week 12 (inpatient: ICP‐TAU odds ratio = −0.11; 95% CI: −0.42, 0.19; LTCH: ICP‐TAU odds ratio = 0.02; 95% CI: −0.29, 0.33). Among the inpatients, post‐hoc analyses showed a significantly lower use of polypharmacy in the ICP than in the TAU group at week 1 (ICP‐TAU odds ratio = −0.26; 95% CI −0.45, −0.06), week 3 (ICP‐TAU odds ratio = −0.38; 95% CI: −0.63, −0.12), and week 4 (ICP‐TAU odds ratio = −0.4; 95% CI: −0.69, −0.10). Among the LTCH residents, post‐hoc analyses showed a significantly lower use of polypharmacy in the ICP than the TAU group at week 1 (ICP‐TAU odds ratio = −0.14; 95% CI: −0.26, −0.02) and week 3 (ICP‐TAU odds ratio = −0.24; 95% CI: −0.39, −0.08) (Figure 3).

**TABLE 2B:** Overall Wald tests from generalized estimating equations for polypharmacy by setting (inpatient vs LTCH).

Covariate	Inpatient			LTCH		
	Wald *χ* ^2^	df	*p* value	Wald *χ* ^2^	df	*p* value
Age	0.012	1	0.914	1.732	1	0.188
Gender	0.003	1	0.954	0.375	1	0.540
Baseline dementia severity	0.325	1	0.569	0.173	1	0.677
Treatment group (ICP vs TAU)	4.216	1	0.040	1.386	1	0.239
Time Point	6.938	7	0.435	11.321	7	0.125
Treatment group × Time interaction	15.315	7	0.032	29.989	7	<0.001
Baseline agitation polypharmacy	47.383	1	<0.001	45.718	1	<0.001

Abbreviations: CMAI, Cohen‐Mansfield Agitation Inventory; ICP, Integrated Care Pathway; LTCH, long‐term‐care home; NA, not applicable; TAU, treatment as usual.

In the inpatient setting, the ICP group started the treatment with an average of 1.5 medications per participant (40% polypharmacy), which dropped to an average of 1.3 (33% polypharmacy) at week 1, and ended with an average of 1.9 medications (53% polypharmacy) at week 12, whereas the TAU group started with an average of 1.6 medications per participant (60% polypharmacy), rose to an average of 1.7 (67% polypharmacy) at week 1, and eventually increased to an average of 2.5 medications (70% polypharmacy) at week 12. Among the LTCH residents, the ICP group started the treatment with an average of 1.0 psychotropic medication per participant (28% polypharmacy), fell to an average of 0.8 (20% polypharmacy) at week 1, and ended at an average of 1.0 (25% polypharmacy) at week 12, whereas the TAU group started with an average of 1.0 medications per participant (26% polypharmacy), increased to an average of 1.2 (30% polypharmacy) at week 1, and eventually ended at 1.1 (24% polypharmacy) at week 12. For further details of number of medications used to treat agitation and polypharmacy at each time point, see Table S1. Further details regarding the distribution of psychotropic medication used by class and treatment group are presented in Tables S2 (inpatient) and S3 (LTCH). Finally, doses of medications to treat agitation in both treatment groups are presented in Tables S4 (inpatient) and S5 (LTCH).

In sensitivity analyses, there were no effects of study site on comparison between the ICP and TAU treatment groups across 12 weeks in terms of CMAI or polypharmacy.

### 3.3 Secondary and exploratory outcomes

Tables S6–S9 show a summary of results from linear mixed models for secondary and exploratory outcomes. There were no significant differences between the ICP and TAU treatment groups for changes in NPS as assessed by the NPI‐C at week 3 or week 12 for the inpatients or LTCH residents. There were no differences between the ICP and TAU groups in the number of falls, caregiver burden as assessed by the ZBI, or health‐related quality of life as assessed by the ADRQL in either inpatients or LTCH residents. Survival analysis using CGIC showed no significant differences between the ICP or TAU groups in time to response for agitation in either inpatients or LTCH residents (Figure S2).

### 3.4 Adverse events and withdrawals

Table S10 shows the summary of adverse events among the inpatients and LTCH residents in the ICP and TAU groups. No adverse events were judged to be causally related to the ICP. Among inpatients, the ICP group experienced two deaths (one before starting any study procedures and another due to pneumonia related to COVID‐19), one withdrawal due to change in primary diagnosis, and three withdrawals due to other reasons. Among inpatients, the TAU group experienced three deaths (likely due to stroke, COVID‐19, and acute kidney failure), no withdrawal due to medical instability, and two withdrawals due to other reasons. Among LTCH residents, the ICP group experienced two deaths (both due to terminal dementia), one withdrawal due to medical instability, and one withdrawal due to other reasons. Among LTCH residents, the TAU group experienced three deaths (due to respiratory infection, terminal dementia, and urosepsis), four withdrawals due to medical instability, and no withdrawal due to other reasons (Figure 1). The rate of mild adverse events was numerically higher in the ICP group, driven by a higher number of urinary tract infections (UTIs). Details of all adverse events by body system and medication‐related adverse effects assessed using AIMS, SAS, and BAS are presented in Tables S11 and S12.

## 4 DISCUSSION

This 12‐week multisite, double‐blind RCT compared an ICP with TAU for the treatment of agitation in dementia on inpatient and LTCH settings. It showed no differences in efficacy between the two treatment groups in either setting. Similarly, the psychotropic polypharmacy did not differ at week 12 between the ICP and TAU groups in either setting. However, the rates of polypharmacy were lower in the ICP group at weeks 1, 3, and 4 in the inpatient setting and lower in the ICP group at weeks 1 and 3 in the LTCH setting. Further, the two treatment groups did not differ in terms of overall NPSs, caregiver burden, time to response for agitation, health‐related quality of life, or adverse events.

Treatment of NPSs of dementia remains challenging despite some recent advances.^60^, ^61^, ^62^, ^63^ Some standardized and team‐based approaches have shown promise but with varying degrees of success.^64^, ^65^, ^66^ For example, an intervention aimed at staff education failed to improve outcomes in a large cluster randomized trial.^64^ Another multicomponent intervention aimed at enhancing the use of non‐pharmacological interventions resulted in improved agitation but did not result in any change in medication use.^65^ A guideline‐based intervention that included training on non‐pharmacological and pharmacological interventions reduced agitation and antipsychotic medication use, but it increased the use of certain other psychotropic medications.^66^ In the current study, both the ICP and TAU led to improvements in agitation, and their efficacy did not differ. Given that the TAU was provided on psychiatry inpatient units and in LTCHs affiliated with academic centers, it is possible that these settings offered a higher quality of care than might be expected in community settings not affiliated with academic centers, resulting in no added benefit from the ICP. Also, at least one of the psychiatry inpatient units participating in this study was familiar with the ICP from a prior pilot implementation,^14^ which may have resulted in the application of the ICP principles in the TAU group. Further, we randomized participants to ICP or TAU in the same inpatient units or LTCHs, which could have again resulted in some TAU participants benefitting from the knowledge of the clinical teams regarding the ICP. Future studies should assess adherence to the ICP principles in both the ICP and TAU groups. Achieving comparable efficacy with lower rates of psychotropic polypharmacy could have significant impact with respect to long‐term adverse effects and costs related to the treatment of agitation in dementia.

While there were no differences in psychotropic polypharmacy at later stages of treatment, it is important to understand the trajectories of medication use for agitation in the ICP and TAU groups. The early decrease in the number of psychotropic medications (and rates of polypharmacy) in the ICP group was likely due to the emphasis on comprehensive assessment, medication clean‐up, and behavioral interventions. There was also an increased detection of UTIs in the ICP group that could have prevented inappropriate psychotropic medication use. Nevertheless, the presence of psychotropic medications at week 1 also suggests that some of the medications were started early rather than allowing for more prolonged behavioral interventions following the medication clean‐up. Also, continued emphasis on behavioral interventions beyond the first 3 weeks could have influenced medication use toward the end of the treatment phase. Overall, the lower use of medication in the ICP group than in the TAU group suggests that the ICP may offer an alternative approach to managing agitation that minimizes the use of pharmacological interventions. Given the known risks and adverse effects associated with psychotropic medications in individuals with dementia,^15^, ^67^ our findings have important implications for clinical practice.

We did not observe any significant differences in secondary outcomes such as rates of falls, caregiver burden, or quality of life. Psychotropic polypharmacy among the elderly and particularly those with dementia is well known to be associated with adverse events such as falls.^68^, ^69^, ^70^ However, falls were relatively rare, and the study was not powered to detect differences in rates of falls or other adverse effects between the ICP and TAU groups. Lack of differences in caregiver burden and quality of life measures between the treatment groups in 12 weeks could be because both groups were getting treatment in highly specialized settings, and the differences in these measures due to the effect of a lower use of medication may require a longer duration to become apparent. Thus, future studies should emphasize behavioral interventions over a longer period and study the outcomes related to ICP implementation over a longer time frame.

This study has some limitations. First, we randomized participants within sites, which could have resulted in contamination due to the same treatment teams treating some participants in the ICP group (i.e., while receiving ICP‐based recommendations) and others in the TAU group. Thus, future studies should consider randomizing at the level of sites rather than participants or other innovative pragmatic trial designs such as cluster randomization. Second, the duration of standalone behavioral interventions was set at 3 weeks, which may not be a sufficient duration to realize the full effect of behavioral interventions. However, this relatively short duration was based on pragmatic considerations as clinical teams may find it hard to withhold pharmacotherapy in acute situations, particularly in inpatient settings. Future studies should consider using different durations of standalone behavioral interventions based on the acuity of the clinical presentation. Third, the trial was conducted in psychiatry inpatient units and in LTCHs affiliated with academic centers, so the results may not be generalizable to community settings. Future studies should consider enrolling community hospitals, LTCHs, and outpatient settings that may have greater variability in care and may benefit more from standardized approache using the ICP.

## 5 CONCLUSIONS

This double‐blind RCT comparing an ICP to TAU for the assessment and treatment of agitation due to dementia in inpatient and LTCH settings did not show improved clinical outcomes in the ICP group or reduced psychotropic polypharmacy at the end of the intervention (i.e., 12 weeks). However, while the clinical outcomes were comparable in both treatment groups in both inpatient and LTCH settings, there were lower rates of early psychotropic polypharmacy for agitation in the ICP groups in both settings, indicating that the ICP may reduce polypharmacy without affecting efficacy of treatment for agitation.

## CONFLICT OF INTEREST STATEMENT

The authors declare no conflicts of interest. Author disclosures are available in the Supporting Information.

## CONSENT STATEMENT

All human participants provided informed consent prior to participation in the study.

## Supporting information




Supporting Information

Supporting Information

Supporting Information

Supporting Information

Supporting Information

Supporting Information

Supporting Information

Supporting Information

Supporting Information

Supporting Information

Supporting Information

Supporting Information

Supporting Information

Supporting Information

Supporting Information
