# Supplementary material for: Standardizing care for agitation in Alzheimer's disease, results from a randomized controlled trial of an integrated care pathway versus usual care – the StaN trial
Source: Alzheimers Dement. 2026 Jul 27;22(7):e71610. doi: 10.1002/alz.71610 (PMC13403223; doi:10.1002/alz.71610)
Supplement: Supplementary file 1 — Supporting Information [file ALZ-22-e71610-s009.docx]

**Supplementary Table 1**: Mean (SD) of Total Medications for Agitation and Polypharmacy (%) by Setting and Treatment at Different Time Points

|  | | | | |
| --- | --- | --- | --- | --- |
|  | **Inpatient** | | **LTCH** | |
| Week | **ICP** | **TAU** | **ICP** | **TAU** |
| Week 0 | 1.5 (1.2); 40% | 1.6 (1.1); 61.7% | 1.0 (0.9); 28.3% | 1.0 (0.7); 26.1% |
| Week 1 | 1.3 (1.1); 32.6% | 1.7 (1); 60.9% | 0.8 (0.8); 20% | 1.2 (0.7); 30.2% |
| Week 3 | 1.4 (1); 36.6% | 2.1 (1.2); 72.1% | 0.8 (0.7); 20.5% | 1.2 (0.8); 31.8% |
| Week 4 | 1.5 (1); 37.5% | 2.2 (1.3); 73.8% | 1 (0.8); 25.6% | 1.2 (0.8); 30.2% |
| Week 6 | 1.7 (1.1); 47.4% | 2.2 (1.4); 68.3% | 1 (0.8); 28.6% | 1.3 (0.7); 35.7% |
| Week 8 | 1.8 (1.3); 51.4% | 2.3 (1.5); 66.7% | 1.1 (0.9); 28.2% | 1.2 (0.8); 31.7% |
| Week 10 | 1.9 (1.5); 47.2% | 2.4 (1.3); 73.7% | 1.0 (0.8); 26.8% | 1.2 (0.8); 32.4% |
| Week 12 | 1.9 (1.3); 52.8% | 2.5 (1.5); 70.3% | 1.0 (0.8); 25% | 1.1 (0.8); 24.3% |

**Abbreviations**: ICP = Integrated Care Pathway; TAU = Treatment As Usual; LTCH = Long-Term Care Home. SD = Standard Deviation
