# Supplementary material for: Standardizing care for agitation in Alzheimer's disease, results from a randomized controlled trial of an integrated care pathway versus usual care – the StaN trial
Source: Alzheimers Dement. 2026 Jul 27;22(7):e71610. doi: 10.1002/alz.71610 (PMC13403223; doi:10.1002/alz.71610)
Supplement: Supplementary file 2 — Supporting Information [file ALZ-22-e71610-s002.docx]

**Supplementary Table 2**: Distribution of Psychotropic Medication Use by Class (n, %) Among Inpatient Participants.

| Class of psychotropic medication used for agitation | Baseline | | Week-1 | | Week-3 | | Week-4 | |
| --- | --- | --- | --- | --- | --- | --- | --- | --- |
|  | ICP | TAU | ICP | TAU | ICP | TAU | ICP | TAU |
|  | (N = 45**^1^**) | (N = 47) | (N = 43) | (N = 46) | (N = 41) | (N = 43) | (N = 40) | (N = 42) |
| Antipsychotic | 29 (64.4%) | 30 (63.8%) | 26 (60.5%) | 30 (65.2%) | 30 (73.2%) | 31 (72.1%) | 32 (80%) | 31 (73.8%) |
| Antidepressant | 15 (33.3%) | 21 (44.7%) | 12 (27.9%) | 24 (52.2%) | 9 (22%) | 24 (55.8%) | 8 (20%) | 24 (57.1%) |
| Benzodiazepine | 6 (13.3%) | 2 (4.3%) | 4 (9.3%) | 2 (4.3%) | 4 (9.8%) | 2 (4.7%) | 3 (7.5%) | 3 (7.1%) |
| Mood Stabilizer | 2 (4.4%) | 0 (0%) | 3 (7%) | 0 (0%) | 2 (4.9%) | 3 (7%) | 3 (7.5%) | 3 (7.1%) |
| Others | 9 (20%) | 13 (27.7%) | 7 (16.3%) | 13 (28.3%) | 7 (17.1%) | 14 (32.6%) | 6 (15%) | 14 (33.3%) |

| Class of psychotropic medication used for agitation | Week-6 | | Week-8 | | Week-10 | | Week-12 | |
| --- | --- | --- | --- | --- | --- | --- | --- | --- |
|  | ICP | TAU | ICP | TAU | ICP | TAU | ICP | TAU |
|  | (N = 38) | (N = 41) | (N = 37) | (N = 39) | (N = 36) | (N = 38) | (N = 36) | (N = 37) |
| Antipsychotic | 33 (86.8%) | 30 (73.2%) | 32 (86.5%) | 27 (69.2%) | 30 (83.3%) | 29 (76.3%) | 28 (77.8%) | 28 (75.7%) |
| Antidepressant | 8 (21.1%) | 24 (58.5%) | 8 (21.6%) | 22 (56.4%) | 9 (25%) | 21 (55.3%) | 10 (27.8%) | 23 (62.2%) |
| Benzodiazepine | 3 (7.9%) | 3 (7.3%) | 3 (8.1%) | 3 (7.7%) | 3 (8.3%) | 4 (10.5%) | 3 (8.3%) | 4 (10.8%) |
| Mood Stabilizer | 2 (5.3%) | 4 (9.8%) | 3 (8.1%) | 5 (12.8%) | 6 (16.7%) | 3 (7.9%) | 5 (13.9%) | 3 (8.1%) |
| Others | 7 (18.4%) | 13 (31.7%) | 7 (18.9%) | 14 (35.9%) | 7 (19.4%) | 16 (42.1%) | 7 (19.4%) | 14 (37.8%) |

^1^: One participant was randomized at baseline but withdrew before medication data collection, so their medication data were not included.

**Abbreviations**: ICP = Integrated Care Pathway; TAU = Treatment As Usual; LTCH = Long-Term Care Home.

Psychotropic medication classes were defined as follows:**Antipsychotics**: haloperidol, loxapine, clozapine, olanzapine, zuclopenthixol (Clopixol depot), aripiprazole, brexpiprazole, methotrimeperazine, quetiapine, risperidone. **Antidepressants**: fluoxetine, bupropion, vortioxetine, citalopram hydrobromide, venlafaxine, escitalopram (Cipralex), duloxetine, mirtazapine, sertraline, trazodone. **Benzodiazepines**: clonazepam, lorazepam. **Mood stabilizers**: divalproex, carbamazepine, oxcarbazepine. **Other medications**: gabapentin, pregabalin, nabilone, zopiclone, memantine, cannabis oil, hydromorphone, cyproterone acetate (Androcur), melatonin, dextromethorphan.
