# Supplementary material for: Standardizing care for agitation in Alzheimer's disease, results from a randomized controlled trial of an integrated care pathway versus usual care – the StaN trial
Source: Alzheimers Dement. 2026 Jul 27;22(7):e71610. doi: 10.1002/alz.71610 (PMC13403223; doi:10.1002/alz.71610)
Supplement: Supplementary file 3 — Supporting Information [file ALZ-22-e71610-s001.docx]

**Supplementary Table 3**: Distribution of Psychotropic Medication Use by Class (n, %) Among LTCH Participants

| Class of psychotropic medication used for agitation | Baseline | | Week-1 | | Week-3 | | Week-4 | |
| --- | --- | --- | --- | --- | --- | --- | --- | --- |
|  | ICP | TAU | ICP | TAU | ICP | TAU | ICP | TAU |
|  | (N = 46) | (N = 46) | (N = 45) | (N = 43) | (N = 44) | (N = 44) | (N = 43) | (N = 43) |
| Antipsychotic | 18 (39.1%) | 20 (43.5%) | 14 (31.1%) | 24 (55.8%) | 19 (43.2%) | 26 (59.1%) | 22 (51.2%) | 25 (58.1%) |
| Antidepressant | 23 (50%) | 21 (45.7%) | 20 (44.4%) | 20 (46.5%) | 15 (34.1%) | 20 (45.5%) | 15 (34.9%) | 19 (44.2%) |
| Benzodiazepine | 1 (2.2%) | 1 (2.2%) | 0 (0%) | 1 (2.3%) | 1 (2.3%) | 1 (2.3%) | 0 (0%) | 1 (2.3%) |
| Mood Stabilizer | 1 (2.2%) | 0 (0%) | 1 (2.2%) | 0 (0%) | 1 (2.3%) | 0 (0%) | 1 (2.3%) | 0 (0%) |
| Others | 3 (6.5%) | 1 (2.2%) | 1 (2.2%) | 1 (2.3%) | 0 (0%) | 2 (4.5%) | 0 (0%) | 2 (4.7%) |

| Class of psychotropic medication used for agitation | Week -6 | | Week -8 | | Week-10 | | Week-12 | |
| --- | --- | --- | --- | --- | --- | --- | --- | --- |
|  | ICP | TAU | ICP | TAU | ICP | TAU | ICP | TAU |
|  | (N = 42) | (N = 42) | (N = 40) | (N = 41) | (N = 41) | (N = 37) | (N = 40) | (N = 37) |
| Antipsychotic | 26 (61.9%) | 27 (64.3%) | 24 (60%) | 25 (61%) | 24 (58.5%) | 24 (64.9%) | 25 (62.5%) | 20 (54.1%) |
| Antidepressant | 12 (28.6%) | 19 (45.2%) | 12 (30%) | 18 (43.9%) | 11 (26.8%) | 16 (43.2%) | 11 (27.5%) | 15 (40.5%) |
| Benzodiazepine | 0 (0%) | 1 (2.4%) | 1 (2.5%) | 0 (0%) | 1 (2.4%) | 0 (0%) | 0 (0%) | 0 (0%) |
| Mood Stabilizer | 1 (2.4%) | 0 (0%) | 1 (2.5%) | 0 (0%) | 1 (2.4%) | 0 (0%) | 1 (2.5%) | 0 (0%) |
| Others | 0 (0%) | 2 (4.8%) | 0 (0%) | 2 (4.9%) | 0 (0%) | 2 (5.4%) | 0 (0%) | 2 (5.4%) |

**Abbreviations**: ICP = Integrated Care Pathway; TAU = Treatment As Usual; LTCH = Long-Term Care Home.

Psychotropic medication classes were defined as follows: **Antipsychotics**: haloperidol, loxapine, clozapine, olanzapine, zuclopenthixol (Clopixol depot), aripiprazole, brexpiprazole, methotrimeperazine, quetiapine, risperidone. **Antidepressants**: fluoxetine, bupropion, vortioxetine, citalopram hydrobromide, venlafaxine, escitalopram (Cipralex), duloxetine, mirtazapine, sertraline, trazodone. **Benzodiazepines**: clonazepam, lorazepam. **Mood stabilizers**: divalproex, carbamazepine, oxcarbazepine. **Other medications**: gabapentin, pregabalin, nabilone, zopiclone, memantine, cannabis oil, hydromorphone, cyproterone acetate (Androcur), melatonin, dextromethorphan.
