# Supplementary material for: Standardizing care for agitation in Alzheimer's disease, results from a randomized controlled trial of an integrated care pathway versus usual care – the StaN trial
Source: Alzheimers Dement. 2026 Jul 27;22(7):e71610. doi: 10.1002/alz.71610 (PMC13403223; doi:10.1002/alz.71610)
Supplement: Supplementary file 4 — Supporting Information [file ALZ-22-e71610-s004.docx]

**Supplementary Table 4**: Mean (SD) Psychotropic Medication Doses Per Day by Treatment Group (ICP vs TAU) at Baseline and Week 12 Among Inpatient Participants

| Class | Medication | Baseline | | Week-12 | |
| --- | --- | --- | --- | --- | --- |
|  |  | **ICP** | **TAU** | **ICP** | **TAU** |
| Antidepressant | Citalopram - mg | 20 (17.32) | 15 (6.45) | 26.67 (11.55) | 17.5 (5) |
|  | Fluoxetine - mg | 20 (NA) | 20 (NA) | 20 (NA) | 20 (NA) |
|  | Mirtazapine - mg | 22.5 (15) | 18.75 (15.91) | 22.5 (21.21) | 22.5 (7.5) |
|  | Sertraline - mg | 106.25 (65.12) | 58.33 (38.19) | 65 (22.36) | 87.5 (88.39) |
|  | Trazodone - mg | 95 (44.52) | 74.22 (43.89) | 90.91 (62.52) | 90 (83.35) |
| Antipsychotic | Aripiprazole - mg | 8.33 (1.44) | 10 (NA) | 9.28 (3.46) | 6.67 (4.16) |
|  | Loxapine - mg | 2.5 (NA) | 31.25 (26.52) | 2.5 (NA) | 28.12 (25.28) |
|  | Olanzapine - mg | 7.68 (5.37) | 8.8 (4.06) | 10.42 (3.15) | 5.94 (3.19) |
|  | Quetiapine - mg | 153.57 (107.46) | 129.25 (133.5) | 129.08 (95.78) | 172.73 (164.11) |
|  | Risperidone - mg | 1.32 (0.97) | 1.48 (0.93) | 1.19 (0.72) | 1.36 (0.64) |
| Benzodiazepine | Clonazepam - mg | 0.25 (0) | 0.62 (0.18) | 0.25 (NA) | 0.5 (NA) |
|  | Lorazepam - mg | 1.25 (1.19) | 0.5 (0) | 0.42 (0.14) | 0.58 (0.38) |
| Other | Gabapentin - mg | 250 (70.71) | 700 (NA) | 520 (349.28) | 933.33 (520.26) |
|  | Hydromorphone - mg | 5 (5.66) | 4.5 (3.77) | 5 (4) | 3.75 (2.06) |
|  | Melatonin - mg | 5.44 (2.35) | 5.67 (1.8) | 5 (2.38) | 5.57 (1.13) |
|  | Memantine - mg | 10 (0) | 15 (5.35) | 10 (NA) | 12 (7.58) |
|  | Pregabalin - mg | 183.33 (80.1) | 172.73 (109.8) | 300 (141.42) | 196.43 (188.43) |

**Abbreviations**: ICP = Integrated Care Pathway; TAU = Treatment As Usual; LTCH = Long-Term Care Home. SD = Standard Deviation.

Standard deviation is reported as NA when only one participant was available in the corresponding group, precluding estimation of variability. An SD of 0 indicates no variability, with all participants in the group receiving identical doses. The ICP group also includes some medications which are not part of the ICP recommendation due to clinical teams prescribing medications outside of the ICP in some cases.
