# Supplementary material for: Standardizing care for agitation in Alzheimer's disease, results from a randomized controlled trial of an integrated care pathway versus usual care – the StaN trial
Source: Alzheimers Dement. 2026 Jul 27;22(7):e71610. doi: 10.1002/alz.71610 (PMC13403223; doi:10.1002/alz.71610)
Supplement: Supplementary file 5 — Supporting Information [file ALZ-22-e71610-s014.docx]

**Supplementary Table 5**: Mean (SD) Psychotropic Medication Doses by Treatment Group (ICP vs TAU) at Baseline and Week 12 Among LTCH Participants.

| Class | Medication | Baseline | | Week-12 | |
| --- | --- | --- | --- | --- | --- |
|  |  | **ICP** | **TAU** | **ICP** | **TAU** |
| Antidepressant | Citalopram - mg | 15 (5.77) | 13.33 (5.16) | 15 (5.77) | 12.5 (5) |
|  | Duloxetine - mg | 30 (NA) | 40 (17.32) | 30 (NA) | 40 (17.32) |
|  | Escitalopram - mg | 9.29 (1.89) | 12.5 (5) | 8.33 (2.89) | 10 (0) |
|  | Mirtazapine - mg | 25 (7.75) | 12.86 (8.35) | 24.38 (11.25) | 16.25 (14.56) |
|  | Sertraline - mg | 91.67 (40.82) | 50 (0) | 112.5 (49.37) | 50 (0) |
|  | Trazodone - mg | 81.88 (52.17) | 43.35 (27.87) | 79.17 (52.04) | 48.75 (29.14) |
| Antipsychotic | Aripiprazole - mg | 5 (NA) | 10 (NA) | 8.5 (2.24) | 3.75 (1.77) |
|  | Quetiapine - mg | 116.67 (84.29) | 126.94 (111.54) | 90.62 (87.56) | 105.83 (79.63) |
|  | Risperidone - mg | 0.51 (0.34) | 1.81 (2.84) | 0.75 (0.43) | 1.98 (3.04) |
| Other | Gabapentin - mg | 533.33 (404.15) | 900 (NA) | 900 (NA) | 900 (NA) |
|  | Hydromorphone - mg | 2 (NA) | 4.83 (6.25) | 0.75 (NA) | 2.25 (2.47) |
|  | Melatonin - mg | 5 (2.19) | 4 (1.41) | 5.5 (2.52) | 3 (NA) |
|  | Memantine - mg | 10 (0) | 16.67 (5.77) | 10 (0) | 15 (7.07) |

**Abbreviations**: ICP = Integrated Care Pathway; TAU = Treatment As Usual; LTCH = Long-Term Care Home. SD = Standard Deviation.

Standard deviation is reported as NA when only one participant was available in the corresponding group, precluding estimation of variability. An SD of 0 indicates no variability, with all participants in the group receiving identical doses.
