# Supplementary material for: Standardizing care for agitation in Alzheimer's disease, results from a randomized controlled trial of an integrated care pathway versus usual care – the StaN trial
Source: Alzheimers Dement. 2026 Jul 27;22(7):e71610. doi: 10.1002/alz.71610 (PMC13403223; doi:10.1002/alz.71610)
Supplement: Supplementary file 6 — Supporting Information [file ALZ-22-e71610-s011.docx]

**Supplementary Table 6**. Type III Tests of Fixed Effects from Linear Mixed Models for Neuropsychiatric Inventory- Clinical (NPI-C) Agitation/Aggression Domain Score.

| Covariate | Inpatient | | | | LTCH | | | |
| --- | --- | --- | --- | --- | --- | --- | --- | --- |
|  | Numerator df | Denominator df | F statistic | p-value | Numerator df | Denominator df | F statistic | p-value |
| Age | 1 | 110.523 | <0.001 | 0.998 | 1 | 130.629 | 2.059 | 0.154 |
| Gender | 1 | 112.907 | 0.208 | 0.650 | 1 | 130.096 | 0.171 | 0.680 |
| Baseline Dementia Severity | 1 | 116.864 | 0.115 | 0.736 | 1 | 121.720 | 0.303 | 0.583 |
| Treatment Group (ICP vs TAU) | 1 | 109.030 | 0.465 | 0.497 | 1 | 120.615 | 0.208 | 0.649 |
| Time Point | 2 | 179.395 | 7.413 | <0.001 | 2 | 191.281 | 10.500 | <0.001 |
| Treatment Group × Time Interaction | 2 | 179.204 | 3.584 | 0.030 | 2 | 191.254 | 0.368 | 0.693 |
| Baseline NPI-C Agitation and Aggression Score (Log-transformed) | 1 | 111.706 | 129.778 | <0.001 | 1 | 123.344 | 353.674 | <0.001 |

**Abbreviations**: ICP = Integrated Care Pathway; TAU = Treatment As Usual; LTCH = Long-Term Care Home. df = Degrees of Freedom
