# Supplementary material for: Standardizing care for agitation in Alzheimer's disease, results from a randomized controlled trial of an integrated care pathway versus usual care – the StaN trial
Source: Alzheimers Dement. 2026 Jul 27;22(7):e71610. doi: 10.1002/alz.71610 (PMC13403223; doi:10.1002/alz.71610)
Supplement: Supplementary file 7 — Supporting Information [file ALZ-22-e71610-s008.docx]

**Supplementary Table 7**. Type III Tests of Fixed Effects from Linear Mixed Models for Neuropsychiatric Inventory (NPI_C) Total Score

| Covariate | Inpatient | | | | LTCH | | | |
| --- | --- | --- | --- | --- | --- | --- | --- | --- |
|  | Numerator df | Denominator df | F statistic | p-value | Numerator df | Denominator df | F statistic | p-value |
| Age | 1 | 107.954 | 0.069 | 0.793 | 1 | 136.518 | 2.477 | 0.118 |
| Gender | 1 | 111.754 | 2.047 | 0.155 | 1 | 136.603 | 0.219 | 0.641 |
| Baseline Dementia Severity | 1 | 113.065 | 0.672 | 0.414 | 1 | 128.906 | 0.293 | 0.589 |
| Treatment Group (ICP vs TAU) | 1 | 106.755 | 0.613 | 0.435 | 1 | 126.761 | 0.099 | 0.753 |
| Time Point | 2 | 164.162 | 14.108 | <0.001 | 2 | 194.195 | 11.647 | <0.001 |
| Treatment Group × Time Interaction | 2 | 163.998 | 2.262 | 0.107 | 2 | 194.217 | 0.158 | 0.854 |
| Baseline NPI-C Total Score (Log-transformed) | 1 | 108.661 | 113.830 | <0.001 | 1 | 127.829 | 294.682 | <0.001 |

**Abbreviations**: ICP = Integrated Care Pathway; TAU = Treatment As Usual; LTCH = Long-Term Care Home. df = Degrees of Freedom
