# Supplementary material for: Standardizing care for agitation in Alzheimer's disease, results from a randomized controlled trial of an integrated care pathway versus usual care – the StaN trial
Source: Alzheimers Dement. 2026 Jul 27;22(7):e71610. doi: 10.1002/alz.71610 (PMC13403223; doi:10.1002/alz.71610)
Supplement: Supplementary file 8 — Supporting Information [file ALZ-22-e71610-s015.docx]

**Supplementary Table 8**. Type III Tests of Fixed Effects from Linear Mixed Models for Zarit Burden Interview (ZBI).

| Covariate | Inpatient | | | | LTCH | | | |
| --- | --- | --- | --- | --- | --- | --- | --- | --- |
|  | Numerator df | Denominator df | F statistic | p-value | Numerator df | Denominator df | F statistic | p-value |
| Age | 1 | 53.881 | 0.110 | 0.741 | 1 | 63.629 | 4.853 | 0.031 |
| Gender | 1 | 57.511 | 0.350 | 0.557 | 1 | 63.431 | 0.019 | 0.890 |
| Baseline Dementia Severity | 1 | 55.799 | 2.837 | 0.098 | 1 | 61.303 | 0.052 | 0.821 |
| Treatment Group (ICP vs TAU) | 1 | 57.113 | 0.981 | 0.326 | 1 | 61.868 | 0.306 | 0.582 |
| Time Point | 2 | 72.396 | 3.776 | 0.028 | 2 | 115.681 | 3.760 | 0.026 |
| Treatment Group × Time Interaction | 2 | 71.923 | 1.388 | 0.256 | 2 | 115.713 | 1.392 | 0.253 |
| Baseline ZBI Score (Log-transformed) | 1 | 61.816 | 31.135 | <0.001 | 1 | 68.768 | 517.202 | <0.001 |

**Abbreviations**: ICP = Integrated Care Pathway; TAU = Treatment As Usual; LTCH = Long-Term Care Home. df = Degrees of Freedom
