# Supplementary material for: Standardizing care for agitation in Alzheimer's disease, results from a randomized controlled trial of an integrated care pathway versus usual care – the StaN trial
Source: Alzheimers Dement. 2026 Jul 27;22(7):e71610. doi: 10.1002/alz.71610 (PMC13403223; doi:10.1002/alz.71610)
Supplement: Supplementary file 9 — Supporting Information [file ALZ-22-e71610-s006.docx]

**Supplementary Table 9.** Type III Tests of Fixed Effects from Linear Mixed Models for Alzheimer’s Disease–Related Quality of Life (ADRQL).

| Covariate | Inpatient | | | | LTCH | | | |
| --- | --- | --- | --- | --- | --- | --- | --- | --- |
|  | Numerator df | Denominator df | F statistic | p-value | Numerator df | Denominator df | F statistic | p-value |
| Age | 1 | 97.313 | 1.053 | 0.307 | 1 | 110.526 | 0.377 | 0.541 |
| Gender | 1 | 99.569 | 0.614 | 0.435 | 1 | 114.944 | 0.308 | 0.580 |
| Baseline Dementia Severity | 1 | 97.881 | 0.221 | 0.639 | 1 | 112.179 | 1.098 | 0.297 |
| Treatment Group (ICP vs TAU) | 1 | 102.177 | 0.381 | 0.539 | 1 | 110.734 | 0.001 | 0.970 |
| Time Point | 2 | 156.511 | 0.816 | 0.444 | 2 | 173.692 | 0.519 | 0.596 |
| Treatment Group × Time Interaction | 2 | 156.467 | 1.298 | 0.276 | 2 | 173.791 | 3.886 | 0.022 |
| Baseline ADRQL Total Score Percentage | 1 | 102.863 | 253.950 | <0.001 | 1 | 120.655 | 222.471 | <0.001 |

**Abbreviations**: ICP = Integrated Care Pathway; TAU = Treatment As Usual; LTCH = Long-Term Care Home. df = Degrees of Freedom
