# Supplementary material for: Standardizing care for agitation in Alzheimer's disease, results from a randomized controlled trial of an integrated care pathway versus usual care – the StaN trial
Source: Alzheimers Dement. 2026 Jul 27;22(7):e71610. doi: 10.1002/alz.71610 (PMC13403223; doi:10.1002/alz.71610)
Supplement: Supplementary file 10 — Supporting Information [file ALZ-22-e71610-s007.docx]

**Supplementary Table 10**. Summary of Serious Adverse Events in each Setting (Inpatient and LTCH) and Treatment Group (ICP and TAU).

| Serious Adverse Events [Count (%)] | | | | | |
| --- | --- | --- | --- | --- | --- |
|  | **Inpatient** | | | **LTCH** | |
|  | **ICP (n=46)** | **TAU (n=47)** | **ICP (n=46)** | | **TAU (n=46)** |
| Abnormal Blood Counts | 1 (2.2%) | 0 (0%) | 0 (0%) | | 0 (0%) |
| Abnormal Kidney Function | 0 (0%) | 1 (2.1%) | 0 (0%) | | 2 (4.4%) |
| Cardiovascular | 2 (4.4%) | 0 (0%) | 1 (2.2%) | | 1 (2.2%) |
| COVID-19 | 1 (2.2%) | 1 (2.1%) | 0 (0%) | | 0 (0%) |
| Delirium | 1 (2.2%) | 0 (0%) | 0 (0%) | | 0 (0%) |
| Dermatological | 0 (0%) | 0 (0%) | 0 (0%) | | 1 (2.2%) |
| Fever | 1 (2.2%) | 0 (0%) | 0 (0%) | | 0 (0%) |
| Gastrointestinal System | 0 (0%) | 0 (0%) | 0 (0%) | | 2 (4.4%) |
| Emergency Dept Visit (Unclear) | 0 (0%) | 0 (0%) | 0 (0%) | | 0 (0%) |
| Musculoskeletal Injury | 0 (0%) | 0 (0%) | 1 (2.2%) | | 0 (0%) |
| Respiratory | 0 (0%) | 1 (2.1%) | 1 (2.2%) | | 0 (0%) |
| Stroke | 0 (0%) | 0 (0%) | 0 (0%) | | 1 (2.2%) |
| Urinary | 0 (0%) | 1 (2.1%) | 0 (0%) | | 1 (2.2%) |

**Abbreviations**: ICP = Integrated Care Pathway; TAU = Treatment As Usual; LTCH = Long-Term Care Home.
