# Supplementary material for: Standardizing care for agitation in Alzheimer's disease, results from a randomized controlled trial of an integrated care pathway versus usual care – the StaN trial
Source: Alzheimers Dement. 2026 Jul 27;22(7):e71610. doi: 10.1002/alz.71610 (PMC13403223; doi:10.1002/alz.71610)
Supplement: Supplementary file 11 — Supporting Information [file ALZ-22-e71610-s013.docx]

**Supplementary Table 11:** Summary of Adverse Events in each Setting (Inpatient and LTCH) and Treatment Group (ICP and TAU)

| Adverse Events [Count (%)] | | | | | |
| --- | --- | --- | --- | --- | --- |
| Events Severity | **Event Type** | **Inpatient** | | **LTCH** | |
|  |  | ICP (n=46) | TAU (n=47) | ICP (n=46) | TAU (n=46) |
| Mild | Abnormal Blood Counts | 1 (2.2%) | 1 (2.1%) | 0 (0%) | 0 (0%) |
|  | Abnormal Kidney Function | 0 (0%) | 1 (2.1%) | 0 (0%) | 1 (2.2%) |
|  | Bleeding | 0 (0%) | 2 (4.3%) | 0 (0%) | 0 (0%) |
|  | Cardiovascular | 1 (2.2%) | 0 (0%) | 1 (2.2%) | 0 (0%) |
|  | COVID-19 | 2 (4.4%) | 2 (4.3%) | 3 (6.5%) | 0 (0%) |
|  | Dermatological | 1 (2.2%) | 0 (0%) | 3 (6.5%) | 4 (8.7%) |
|  | Dizziness | 1 (2.2%) | 0 (0%) | 0 (0%) | 0 (0%) |
|  | Edema | 0 (0%) | 1 (2.1%) | 0 (0%) | 0 (0%) |
|  | Eye | 2 (4.4%) | 0 (0%) | 2 (4.4%) | 1 (2.2%) |
|  | Fever | 0 (0%) | 0 (0%) | 1 (2.2%) | 0 (0%) |
|  | Gastrointestinal System | 3 (6.5%) | 2 (4.3%) | 3 (6.5%) | 1 (2.2%) |
|  | Musculoskeletal Injury | 0 (0%) | 1 (2.1%) | 0 (0%) | 1 (2.2%) |
|  | Teeth | 0 (0%) | 0 (0%) | 1 (2.2%) | 0 (0%) |
|  | Respiratory | 1 (2.2%) | 2 (4.3%) | 3 (6.5%) | 2 (4.4%) |
|  | Seizure | 1 (2.2%) | 0 (0%) | 0 (0%) | 0 (0%) |
|  | Urinary | 5 (10.9%) | 1 (2.1%) | 9 (19.6%) | 0 (0%) |
| Moderate | Abnormal Kidney Function | 0 (0%) | 1 (2.1%) | 0 (0%) | 0 (0%) |
|  | Bleeding | 1 (2.2%) | 0 (0%) | 0 (0%) | 0 (0%) |
|  | COVID-19 | 0 (0%) | 0 (0%) | 1 (2.2%) | 0 (0%) |
|  | Dermatological | 0 (0%) | 1 (2.1%) | 2 (4.4%) | 0 (0%) |
|  | Edema | 0 (0%) | 1 (2.1%) | 0 (0%) | 0 (0%) |
|  | Gastrointestinal System | 0 (0%) | 1 (2.1%) | 1 (2.2%) | 3 (6.5%) |
|  | Head Injury | 0 (0%) | 1 (2.1%) | 0 (0%) | 0 (0%) |
|  | Musculoskeletal Injury | 0 (0%) | 2 (4.3%) | 1 (2.2%) | 1 (2.2%) |
|  | Emergency Department Visit (Unclear) | 0 (0%) | 1 (2.1%) | 0 (0%) | 0 (0%) |
|  | Respiratory | 0 (0%) | 1 (2.1%) | 2 (4.4%) | 0 (0%) |
|  | Seizure | 1 (2.2%) | 1 (2.1%) | 0 (0%) | 0 (0%) |
|  | Urinary | 1 (2.2%) | 0 (0%) | 2 (4.4%) | 0 (0%) |
| Severe | Fever | 1 (2.2%) | 0 (0%) | 0 (0%) | 1 (2.2%) |
|  | Gastrointestinal System | 0 (0%) | 0 (0%) | 0 (0%) | 0 (0%) |
|  | Musculoskeletal Injury | 0 (0%) | 1 (2.1%) | 0 (0%) | 0 (0%) |
|  | Respiratory | 0 (0%) | 1 (2.1%) | 0 (0%) | 0 (0%) |

**Abbreviations**: ICP = Integrated Care Pathway; TAU = Treatment As Usual; LTCH = Long-Term Care Home.
