# Supplementary material for: Standardizing care for agitation in Alzheimer's disease, results from a randomized controlled trial of an integrated care pathway versus usual care – the StaN trial
Source: Alzheimers Dement. 2026 Jul 27;22(7):e71610. doi: 10.1002/alz.71610 (PMC13403223; doi:10.1002/alz.71610)
Supplement: Supplementary file 12 — Supporting Information [file ALZ-22-e71610-s005.docx]

**Supplementary Table 12**. Abnormal Involuntary Movement (AIMS), Parkinsonism (SAS), and Akathisia (BARS) Scores in each Setting (Inpatient and LTCH), Treatment Group (ICP and TAU), and Time Point.

| Setting | Treatment | Assessment | Week 0  (Mean ± SD [n]) | Week 3  (Mean ± SD [n]) | Week 12  (Mean ± SD [n]) |
| --- | --- | --- | --- | --- | --- |
| Inpatient | **ICP** | BARS | 1.0 ±1.4 [27] | 1.1 ±1.4 [25] | 0.7±1.1 [19] |
|  |  | AIMS | 0.5±1.0 [27] | 0.4±0.8 [26] | 0.5±0.8 [19] |
|  |  | SAS | 4.1±4.6 [27] | 4.2±3.4 [24] | 4.7±3.8 [19] |
|  | **TAU** | BARS | 0.8±1.2 [26] | 0.6±1.1 [23] | 0.5±0.9 [18] |
|  |  | AIMS | 0.3±0.9 [25] | 0.4±0.7 [23] | 0.3±0.8 [18] |
|  |  | SAS | 5.2±5.0 [26] | 4.7±3.8 [22] | 5.8±5.3 [18] |
| LTCH | **ICP** | BARS | 0.2±0.5 [31] | 0.3±0.9 [27] | 0.5±0.9 [22] |
|  |  | AIMS | 0.4±0.8 [31] | 0.3±0.6 [27] | 0.4±0.6 [22] |
|  |  | SAS | 4.0±5.0 [31] | 3.8±5.0 [26] | 4.7±5.7 [22] |
|  | **TAU** | BARS | 0.0±0.2 [31] | 0.0±0.0 [26] | 0.1±0.2 [18] |
|  |  | AIMS | 0.3±0.6 [31] | 0.2±0.5 [26] | 0.2±0.4 [18] |
|  |  | SAS | 3.8±4.9 [31] | 4.3±4.6 [24] | 3.5±2.8 [18] |

**Abbreviations**: ICP = Integrated Care Pathway; TAU = Treatment As Usual; LTCH = Long-Term Care Home.
