# Supplementary material for: Standardizing care for agitation in Alzheimer's disease, results from a randomized controlled trial of an integrated care pathway versus usual care – the StaN trial
Source: Alzheimers Dement. 2026 Jul 27;22(7):e71610. doi: 10.1002/alz.71610 (PMC13403223; doi:10.1002/alz.71610)
Supplement: Supplementary file 14 — Supporting Information [file ALZ-22-e71610-s003.pdf]

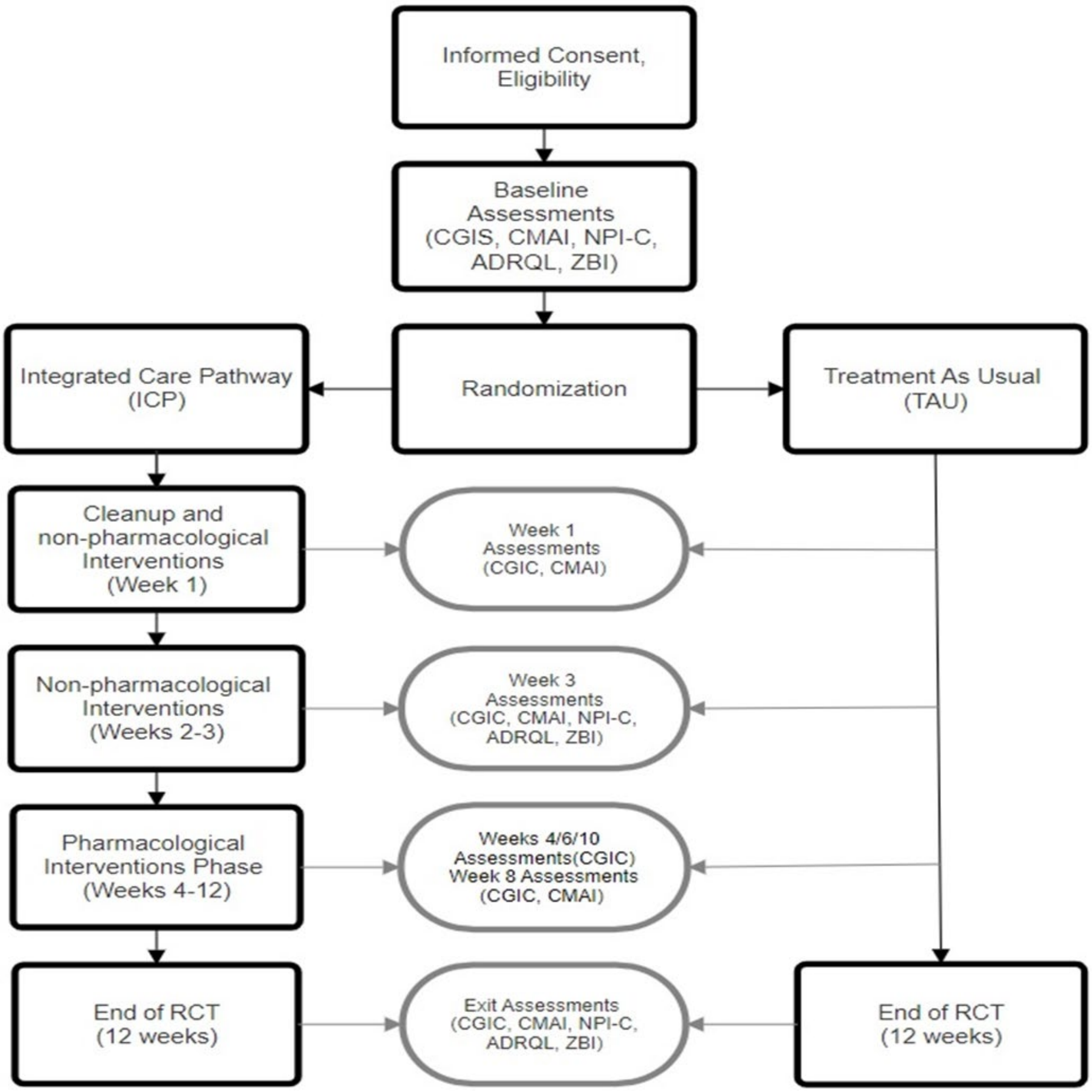

Supplementary Figure 1: Study Design

Abbreviations: ADRQL, Alzheimer's Disease Related Quality of Life Scale; CGIC, Clinical Global Impression of Change Scale; CGIS, Clinical Global Impression–Severity Scale; CMAI, Cohen–Mansfield Agitation Inventory; ICP, Integrated Care Pathway; NPI-C, Neuropsychiatric Inventory–Clinician Rating Scale; RCT, randomized controlled trial; TAU, treatment-as-usual; ZBI, Zarit Caregiver Burden Interview
