# Supplementary material for: Standardizing care for agitation in Alzheimer's disease, results from a randomized controlled trial of an integrated care pathway versus usual care – the StaN trial
Source: Alzheimers Dement. 2026 Jul 27;22(7):e71610. doi: 10.1002/alz.71610 (PMC13403223; doi:10.1002/alz.71610)
Supplement: Supplementary file 15 — Supporting Information [file ALZ-22-e71610-s010.pdf]

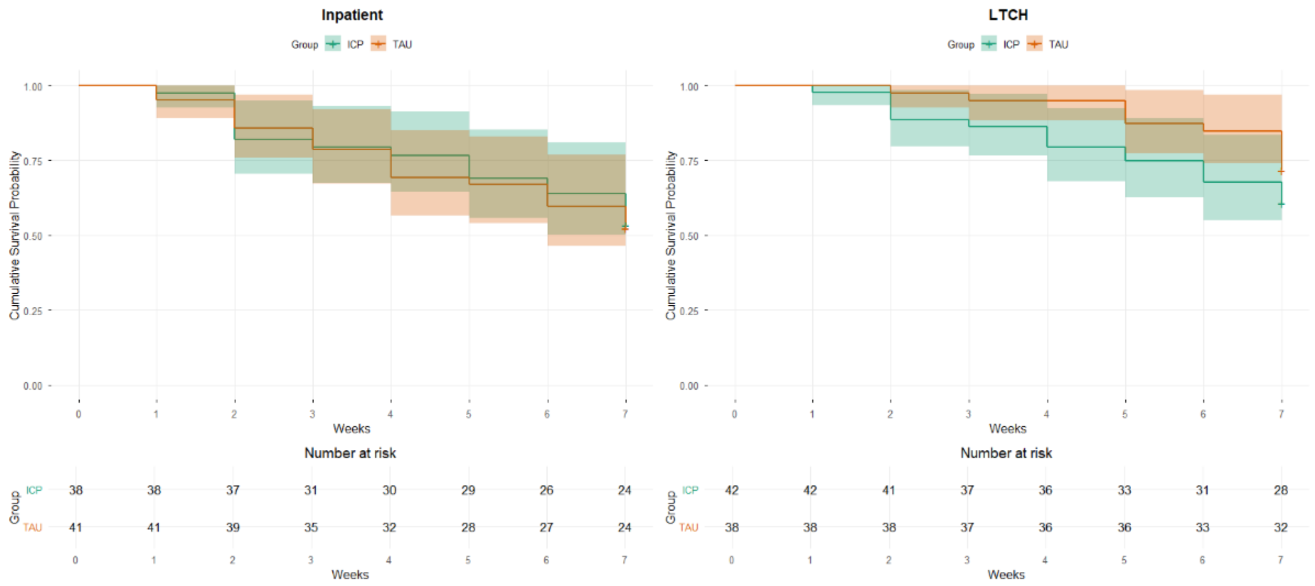

Supplementary Figure 2: Adjusted Survival Curves from Cox Proportional Hazards Models Comparing Clinical Global Impressions of Change Outcomes between ICP and TAU in Inpatient and LTCH Settings.

Note. Two participants in the inpatient ICP arm and one participant in the inpatient TAU arm were excluded due to unavailable baseline FAST stage data. One participant in the LTCH TAU arm was excluded because survival outcome data were missing.
